# Rapid implementation of mobile technology for real-time epidemiology of COVID-19

**DOI:** 10.1126/science.abc0473

**Published:** 2020-05-05

**Authors:** David A. Drew, Long H. Nguyen, Claire J. Steves, Cristina Menni, Maxim Freydin, Thomas Varsavsky, Carole H. Sudre, M. Jorge Cardoso, Sebastien Ourselin, Jonathan Wolf, Tim D. Spector, Andrew T. Chan, COPE Consortium

**Affiliations:** 1 Clinical and Translational Epidemiology Unit, Massachusetts General Hospital and Harvard Medical School, 55 Fruit St., Boston, MA 02114, USA.; 2 Department of Twin Research and Genetic Epidemiology, King’s College London, Westminster Bridge Road, London SE1 7EH, UK.; 3 Department of Ageing and Health, Guys and St. Thomas’ NHS Foundation Trust, Lambeth Palace Road, London SE1 7EH, UK.; 4 School of Biomedical Engineering & Imaging Sciences, King’s College London, 1 Lambeth Palace Road, London SE1 7EU, UK.; 5 Zoe Global Limited, 164 Westminster Bridge Road, London SE1 7RW, UK.; 6 Department of Immunology and Infectious Diseases, Harvard T.H. Chan School of Public Health, 665 Huntington Ave., Boston, MA 02114, USA.

## Abstract

The rapid pace of the coronavirus disease 2019 (COVID-19) pandemic caused by severe acute respiratory syndrome coronavirus 2 (SARS-CoV-2) presents challenges to the robust collection of population-scale data to address this global health crisis. We established the COronavirus Pandemic Epidemiology (COPE) Consortium to unite scientists with expertise in big data research and epidemiology to develop the COVID Symptom Study, previously known as the COVID Symptom Tracker, mobile application. This application—which offers data on risk factors, predictive symptoms, clinical outcomes, and geographical hotspots—was launched in the United Kingdom on 24 March 2020 and the United States on 29 March 2020 and has garnered more than 2.8 million users as of 2 May 2020. Our initiative offers a proof of concept for the repurposing of existing approaches to enable rapidly scalable epidemiologic data collection and analysis, which is critical for a data-driven response to this public health challenge.

## Mobile symptom tracking

The rapidity with which severe acute respiratory syndrome coronavirus 2 (SARS-CoV-2) spreads through a population is defying attempts at tracking it, and quantitative polymerase chain reaction testing so far has been too slow for real-time epidemiology. Taking advantage of existing longitudinal health care and research patient cohorts, Drew *et al.* pushed software updates to participants to encourage reporting of potential coronavirus disease 2019 (COVID-19) symptoms. The authors recruited about 2 million users (including health care workers) to the COVID Symptom Study (previously known as the COVID Symptom Tracker) from across the United Kingdom and the United States. The prevalence of combinations of symptoms (three or more), including fatigue and cough, followed by diarrhea, fever, and/or anosmia, was predictive of a positive test verification for SARS-CoV-2. As exemplified by data from Wales, United Kingdom, mathematical modeling predicted geographical hotspots of incidence 5 to 7 days in advance of official public health reports.

*Science*, this issue p. 1362

## Teaser

A mobile app, the COVID Symptom Study, offers data on risk factors, early symptoms, clinical outcomes, and geographical hotspots.

The exponentially increasing number of severe acute respiratory syndrome coronavirus 2 (SARS-CoV-2) infections has led to “an urgent need to expand public health activities in order to elucidate the epidemiology of the novel virus and characterize its potential impact” (*1*). Understanding risk factors for infection and predictors of subsequent outcomes is crucial to gain control of the coronavirus disease 2019 (COVID-19) pandemic (*2*). However, the speed at which the pandemic is unfolding poses an unprecedented challenge for the collection of exposure data to characterize the full breadth of disease severity, hampering efforts for timely dissemination of accurate information to affect public health planning and clinical management. Thus, there is an urgent need for an adaptable real-time data-capture platform to rapidly and prospectively collect actionable high-quality data that encompass the spectrum of subclinical and acute presentations and identify disparities in diagnosis, treatment, and clinical outcomes. Addressing this priority will allow for more accurate estimates of disease incidence, inform risk mitigation strategies, facilitate allocation of scarce testing resources, and encourage appropriate quarantine and treatment of those afflicted.

An evolving body of literature suggests that COVID-19 incidence and outcomes vary according to age, sex, race, ethnicity, and underlying health status, with inconsistent evidence suggesting that commonly used medications—such as angiotensin-converting enzyme inhibitors, thiazolidinediones, and ibuprofen—may alter the natural disease course (*3*–*9*). Further, symptoms of COVID-19 vary widely, with fever and dry cough reportedly the most prevalent, though numerous investigations have demonstrated that asymptomatic carriage is a significant determinant of community spread (*5*–*7*, *10*–*13*). In addition, the full spectrum of clinical presentation, which is still being characterized, may differ markedly across patient subgroups. Recent advisories from the American Gastroenterological Association, the American Academy of Otolaryngology–Head and Neck Surgery, and the British Geriatrics Society have emphasized the potential association between COVID-19 infection and previously underappreciated gastrointestinal symptoms (e.g., nausea, anorexia, and diarrhea), loss of taste and/or smell, and common geriatric syndromes (e.g., falls and delirium). The pandemic has considerably outpaced our collective efforts to fully characterize who is most at risk or may suffer the most serious sequelae of infection.

Mobile phone applications and web-based tools facilitate self-guided collection of population-level data at scale (*14*), the results of which can be rapidly redeployed to inform participants of urgent health information (*14*, *15*). Both technologies are particularly advantageous when many individuals are advised to maintain physical distance from others (*16*). Such digital tools have already been applied in more controlled research settings, and these studies benefit from greater lead time for field testing, question curation, and recruitment. Although many digital collection tools for COVID-19 are being developed and launched in the Unites States and abroad (see http://mhealth-hub.org/mhealth-solutions-against-covid-19 for a continuously updated resource list from the European Union and the World Health Organization), including some in partnership with government health agencies such as the Centers for Disease Control and Prevention, most applications have largely been configured to offer a single assessment of symptoms to tailor semipersonalized recommendations for further evaluation. Infectious disease surveillance web-based tools (e.g., http://flunearyou.org) have been rapidly adapted for COVID-19–specific collection (e.g., http://covidnearyou.org). Alternatively, web portals have been developed for researchers to report patient-level information on behalf of participants already enrolled in clinical registries (e.g., ccc19.org). Integration with approaches that use remote data capture (e.g., wearable technology or symptom checkers such as real-time reporting thermometers) is also being considered. Although these approaches offer critical public health insights, they are often not tailored for the type of scalable longitudinal data capture that epidemiologists need to perform comprehensive, well-powered investigations.

To meet this challenge, we established a multinational collaboration, the COronavirus Pandemic Epidemiology (COPE) Consortium, composed of leading investigators from several large clinical and epidemiological cohort studies. COPE brings together a multidisciplinary team of scientists with expertise in big data research and translational epidemiology to investigate the COVID-19 pandemic in a large and diverse patient population. Several large cohorts have already agreed to join these efforts, including the Nurses’ Health Study series, the Growing Up Today Study (GUTS), the Health Professionals Follow-Up Study (HPFS), TwinsUK, American Cancer Society Cancer Prevention Study 3 (CPS-3), the Multiethnic Cohort Study, the California Teachers Study (CTS), the Black Women’s Health Study (BWHS), the Sister Study, Aspirin in Reducing Events in the Elderly (ASPREE), the Stanford Nutrition Studies, the Gulf Long-term Follow-up (GuLF) Study, the Agricultural Health Study, the National Institute of Environmental Health Sciences Environmental Polymorphisms Registry, the Predicting Progression of Developing Myeloma in a High-Risk Screened Population (PROMISE) study, and the Precursor Crowdsourcing (PCROWD) study. To aid in our data harmonization efforts in the United States, we developed the COVID Symptom Study (previously known as the COVID Symptom Tracker) mobile app with in-kind contributions from Zoe Global Ltd., a digital health care company, and academic scientists from Massachusetts General Hospital and King’s College London. By leveraging the established digital backbone of an application used for personal nutrition studies, the COVID Symptom Study app was launched in the United Kingdom on 24 March 2020 and became available in the United States on 29 March 2020 (https://covid.joinzoe.com/us). The COPE Consortium is committed to the shared international pursuit of combating COVID-19 and has worked with scientific collaborators and thought leaders in real-time epidemiology to prioritize data harmonization and sharing as part of the Coronavirus Census Collective (*17*).

**Fig. 1:**
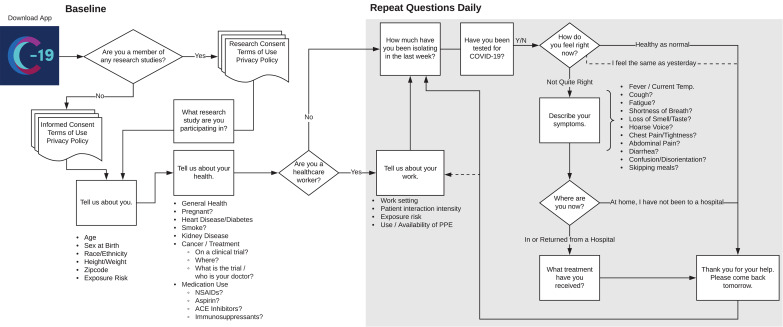
Schematic of the participant workflow. After downloading the COVID Symptom Study app and providing consent, users are prompted to enter baseline demographic and clinical information and are serially queried about new or ongoing symptoms, testing results, and extent of isolation. Health care workers offer additional information about the intensity of their patient interactions, potential exposure to infected patients, and use of PPE. With informed consent, users also participating in a variety of ongoing cohorts or clinical trials (Nurses’ Health Study, TwinsUK, and others) have the option of linking COVID Symptom Study information to their extant research data.

The COVID Symptom Study enables self-reporting of data related to COVID-19 exposure and infections (Fig. 1). On first use, the app queries location, age, and core health risk factors. Daily prompts query for updates on interim symptoms, health care visits, and COVID-19 testing results. For those self-quarantining or seeking health care, the level of intervention and related outcomes are collected. Individuals without obvious symptoms are also encouraged to use the app. Through pushed software updates, we can add or modify questions in real time to test emerging hypotheses about COVID-19 symptoms and treatments. Notably, participants enrolled in ongoing epidemiologic studies, clinical cohorts, or clinical trials can provide informed consent to link survey data collected through the app to their preexisting study cohort data and any relevant biospecimens in a Health Insurance Portability and Accountability Act (HIPAA)–compliant and General Data Protection Regulation (GDPR)–compliant manner. A specific module is also provided for health care workers to determine the intensity and type of their direct patient care experiences, the availability and use of personal protective equipment (PPE), and work-related stress and anxiety.

**Fig. 2:**
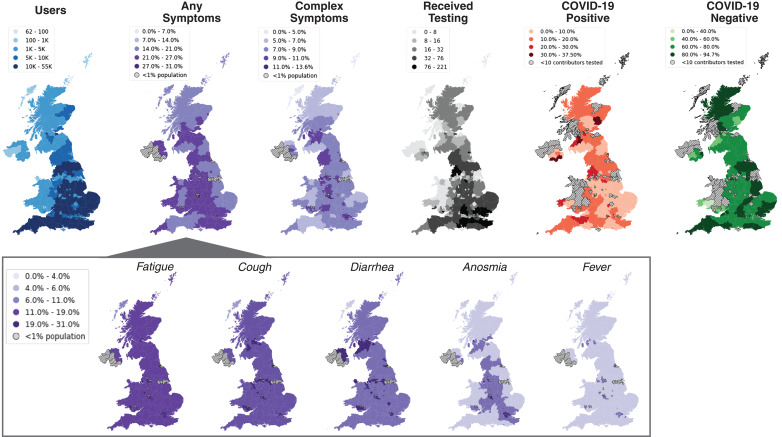
COVID Symptom Study use, reported symptoms, and testing results according to geographic location in the United Kingdom. Between 24 and 29 March 2020, more than 1.6 million unique individuals downloaded the application and shared clinical and demographic information, as well as daily symptoms and high-intensity occupational exposures (blue map). Population density of those presenting with any symptoms (left purple map) varied according to region, with widespread reports of fatigue, cough, and diarrhea, followed by anosmia and, relatively infrequently, fever (inset). Examination of individuals who reported complex symptoms (right purple map), defined as having cough or fever and at least one other symptom (diarrhea, anosmia, or fever), reveals areas that potentially need more testing. For the subset of the population that received a COVID-19 test (black map), areas with larger proportions of positive tests (orange map) appear to coincide with areas in which high proportions of the population reported complex symptoms. By contrast, some areas with low prevalence of complex symptoms have received higher rates of testing and, consequently, more negative tests (green map). This example of real-time visualization of data captured by the COVID Symptom Study may help public health and government officials reallocate resources, identify areas with unmet testing needs, and detect emerging hotspots.

**Fig. 3:**
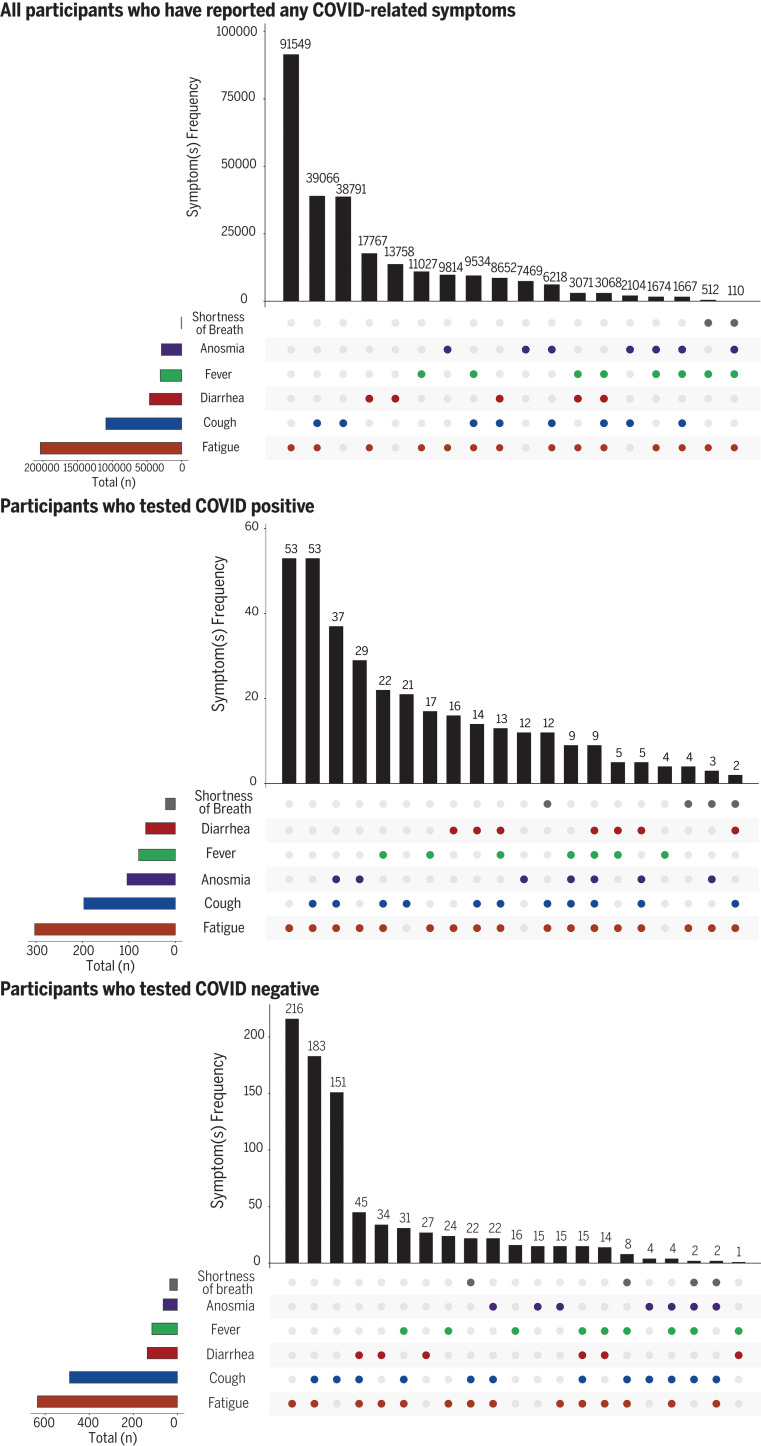
Symptoms reported through the COVID Symptom Study app. By 27 March 2020, 265,851 individuals in the United Kingdom reported any symptom potentially associated with COVID-19 (**top**). Participants provided data on whether they were tested for COVID-19, as well as the test result. 1176 individuals reported having received a COVID-19 test (0.4% of those with symptoms). Symptom frequencies among those who tested positive (**middle**; *n* = 340) versus negative (**bottom**; *n* = 836) are shown.

Through rapid deployment of this tool, we can gain key insights into population dynamics of the disease (Fig. 2). By collecting participant-reported geospatial data, highlighted as a critical need for pandemic epidemiologic research (*15*), we can rapidly identify populations with highly prevalent symptoms in regions that may emerge as outbreak hotspots. An early snapshot of the first 1.6 million users in the United Kingdom over the first 5 days of use confirms the variability in symptoms reported across suspected COVID-19 cases and is useful for generating and testing broader hypotheses. At the time, users had a mean age of 41, ranged from 18 to 90 years old, and were 75% female. Graphic visualization of our initial results (Fig. 3) demonstrates that among those reporting symptoms by 27 March 2020 (*n* = 265,851 individuals), the most common symptoms were fatigue and cough, followed by diarrhea, fever, and anosmia. Shortness of breath was reported relatively rarely. Only 0.4% (*n* = 1176) of individuals reporting possible COVID-19 symptoms reported receiving a quantitative polymerase chain reaction test for COVID-19.

A comparison of symptomatic users who reported receiving a test within the initial launch period generated several hypotheses for future study with the growing dataset. The frequency of cough or fatigue alone or in combination commonly led to testing but was not a particularly accurate predictor of a positive test. Similarly, no individuals who reported diarrhea in the absence of other symptoms tested positive. Notably, more complex presentations with cough and/or fatigue and at least one additional symptom, including less commonly appreciated complaints such as diarrhea and anosmia, appeared to be enriched among those with positive test results relative to those with negative results. In particular, anosmia may be a more predictive symptom, as it was more common than fever in individuals who tested positive. Indeed, in subsequent analyses with a larger sample set, we have shown that anosmia appears to be a strong predictor for COVID-19 (*18*). By contrast, fever alone was not particularly discriminatory. However, when fever was present in combination with less appreciated symptoms, a greater frequency of positive tests was observed. These findings suggest that perhaps individuals with complex or multiple-symptom (three or more) presentations should be prioritized for testing. Concerningly, 20% of individuals reported complex symptoms (cough and/or fatigue plus at least one of anosmia, diarrhea, or fever) but had not yet been tested, representing a substantial population that appears to be at elevated risk for the disease. Additional work is warranted to confirm whether complex or multiple-symptom cases can accurately predict COVID-19 incidence.

**Fig. 4:**
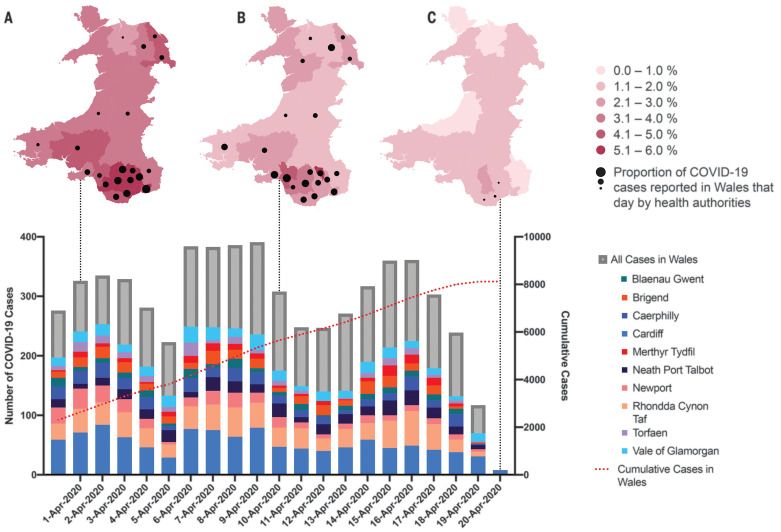
Predicting COVID-19 cases on the basis of real-time symptom reporting in Wales, United Kingdom. This time series (bar graph) displays the number of new confirmed cases (gray bars) reported by the Public Health Wales NHS Trust between 31 March 2020 and 20 April 2020. After 2 April, case numbers appear to have declined through 5 April. However, our symptom-based prediction model (*18*), developed from symptom reports from untested users of the COVID Symptom Study app, showed a high proportion of predicted COVID-19 cases in southern Wales on 1 April [dark red areas in (**A**)]. Six days later, Welsh health authorities reported a subsequent peak in cases over a 4-day period (6 to 9 April), driven primarily by these southern regions (colored bars). By 10 April, new confirmed cases across Wales declined. However, on the basis of reported symptoms (**B**), regions in South Wales still had high predicted levels of COVID-19, which became apparent as a second spike in confirmed cases between 15 and 16 April. As of 20 April (**C**), predicted COVID-19 prevalence across Wales according to symptom reporting appears to be low, which corresponds to a flattening of the cumulative incidence curve. However, several regions in southern Wales still have relatively high reports of symptoms and appear at risk for subsequent cases of COVID-19. Black dots on the maps represent the relative proportion of positive tests reported by health authorities across Wales that day by region. The prediction mapping included data from 1,339,670 users of the COVID Symptom Study on 1 April; 998,244 users on 10 April; and 1,234,918 users on 20 April. Public Health Wales NHS Trust data were current as of 21 April 2020 at 13:00 local time, taken from the “Rapid COVID-19 Virology - Public” dashboard (accessed via https://phw.nhs.wales/), and downloaded on 22 April 2020 at 12:30 p.m. Eastern Standard Time.

Building on these initial findings, our team subsequently developed a weighted prediction model based on the symptoms of more than 2 million individual app users (*18*). By using this prediction model, we demonstrate the potential utility of the COVID Symptom Study app to collect data for long-term studies as well as for immediate public health planning. In southern Wales in the United Kingdom, users reported symptoms that predicted, 5 to 7 days in advance, two spikes in the number of confirmed positive COVID-19 cases reported by public health authorities (Fig. 4). Conversely, a decline in reports of symptoms preceded a drop in confirmed cases by several days. These results demonstrate that this app prospectively captures the dynamics of COVID-19 incidence days in advance of traditional measures, such as positive tests, hospitalizations, or mortality. We are currently planning additional studies using a broadly representative sample of individuals who will undergo uniform COVID-19 testing to further validate our approach to symptom-based modeling of incidence. These data demonstrate compelling evidence for the potential predictive power of our approach, which will improve as more data are collected to inform the model. Further, our data highlight the potential utility of real-time symptom tracking to help guide allocation of resources for testing and treatment as well as recommendations for lockdown or easement in specific areas.

With additional data collection, we will also apply big data approaches (e.g., machine learning) to identify emerging patterns in dynamic settings of exposure, onset of symptoms, disease trajectory, and clinical outcomes. Our launch of the app within several large epidemiology cohorts that have previously gathered longitudinal data on lifestyle, diet and health factors, and genetic information will allow investigation of a much broader range of putative risk factors for COVID-19 outcomes. With additional follow-up, we will also be positioned to investigate long-term effects of COVID-19, including mental health, disability, mortality, and financial outcomes. Mobile technology can also supplement recently launched clinical trials or biobanking protocols already embedded within clinical settings. In collaboration with the Stand Up to Cancer foundation, we have also developed a strategy to track information among individuals living with cancer, including those enrolled in clinical trials. At the Massachusetts General Hospital and Brigham and Women’s Hospital, we are deploying the tool within several clinical studies, centralized biobanking efforts, and health care worker surveillance programs. Health care workers are particularly vulnerable to COVID-19’s effects beyond infection, including work hazards from PPE shortages, emotional stress, and absenteeism. Real-time data generation focused within these populations will be critical to optimally allocate resources to protect our health care workforce and assess its efficacy.

Even so, our approach has limitations. We recognize that a smartphone application does not represent a random sampling of the population. However, this is an inherent limitation of any epidemiologic study that relies on voluntary participation. Our approach has the benefit of allowing rapid deployment across a large cross section of the population during a major public health crisis. With time and continued use, the large number of participants will include a sufficient quantity of users within key subgroups such that we can adjust our methodology for potential sources of confounding. By engaging cohorts with underrepresented populations, such as the BWHS in the United States, we also hope to leverage existing investigator-participant relationships to encourage enrollment of individuals from populations that have traditionally been challenging to recruit. Moreover, by encouraging longitudinal, prospective data collection, we can capture associations based on within-person variation over time, a notable advantage over repeated cross-sectional surveys that introduce considerable between-person variation. In the near future, we hope to release our app as fair-use open source software to facilitate translation and development in other regions. We have begun working with colleagues in Canada, Australia, and Sweden to implement this tool within their countries. We have also developed a practical toolkit to assist clinical researchers with local institutional review board and regulatory approval to facilitate deployment within research studies (www.monganinstitute.org/cope-consortium). This toolkit includes full details of the mobile app’s questions, consent documents, privacy policies, and terms of use. With broader implementation, data generated from the COVID Symptom Study app are increasingly being linked to the public health response within the National Health Service (NHS) in the United Kingdom. The app is endorsed by the Welsh government, NHS Wales, the Scottish government, and NHS Scotland, and our scientific team updates the U.K. chief scientific officer daily. We are working to develop a similar approach in the United States. However, the lack of a national health care system has required a strategy focused on engaging local public health leaders. For example, we have partnered with the University of Texas School of Public Health to conduct statewide surveillance to support public health decision-making, especially as the Texas state government begins softening mitigation strategies.

Our approach demonstrates a proof of concept for rapid repurposing of existing data collection methods to implement scalable real-time collection of population-level data during a fast-moving global health crisis. We call on our colleagues to work with us so that we may deploy all of the tools at our disposal to address this unprecedented public health challenge.

## Supplementary Materials

science.sciencemag.org/content/368/6497/1362/suppl/DC1 Figs. S1 to S9 COPE Consortium Members MDAR Reproducibility Checklist
